# Interrelationship of βeta-2 microglobulin, blood urea nitrogen and creatinine in streptozotocin-induced diabetes mellitus in rabbits

**Published:** 2014

**Authors:** Shahram Javadi, Siamak Asri-Rezaei, Maryam Allahverdizadeh

**Affiliations:** *Department of Clinical Sciences, Faculty of Veterinary Medicine, Urmia University, Urmia, Iran*

**Keywords:** Βeta-2 microglobulin, Diabetes Mellitus, Streptozotocin

## Abstract

Measurement of serum creatinine (Cr) and blood urea nitrogen (BUN) are used as indicators of glomerular filtration rate. The increased levels of these biomarkers are usually detectable at advanced stages of kidney complications. The aim of this study was to find the interrelationship of beta-2 microglobulin (β2M), BUN and Cr in streptozotocin (STZ)-induced diabetes mellitus in rabbits. Diabetes was induced by a single intraperitoneal (IP) injection of 65 mg kg^-1^ of STZ in rabbits. The levels of serum insulin, glucose and three above mentioned biomarkers were measured one day before (day -1) and on days 1-3 after injection of STZ and continued weekly to the end of the experiment (12 weeks). A statistically significant increase of serum β2M, BUN, Cr and glucose levels, and a significant decrease of insulin levels were observed in diabetic animals. However, β2M levels increased as early as one day after STZ injection compared to Cr and BUN that elevated at day two, suggesting a probable diagnostic advantage of β2M over currently used biomarkers in diabetic related kidney complications.

## Introduction

Diabetes mellitus has been considered the most common cause of end-stage renal disease (ESRD) in the United States and Europe, and up to 30% of patients with type I or type II diabetes develop evidence of nephronpathy.^1^ Diabetic nephropathy, which is caused by accumulation of leukocytes in the kidney glomeruli, is characterized by nephrotic syndrome and diffuse glomerulo-sclerosis.^2^ Diabetic nephropathy is diagnosed by a routine urinalysis and by screening for microalbuminuria.^1^

Several studies have demonstrated that tubular involvement may precede glomerular involvement, leading to an increase of various serum and urinary markers, including glomerular-transferrin, fibronectin, beta-2 microglobulin (β2M), retinol binding protein, alpha-1 microglobulin, ephrin-B2, annexin A7, paternally expressed 10 (PEG10), cystatin C, tammhorsfall protein, beta 2 glycoprotein-1, urinary enzymes (N-acetyl-beta-D-glucosaminidase, choline-sterase, gamma glutamyltranspeptidase, alanine amino-peptidase), and tubular brush-border antigen, following diabetic nephropathy.^3^^-^^7^

Beta-2 microglobulin is a low molecular weight protein (11600 Da) found on the surface of lymphocytes and other nucleated cells. Free molecules are also detectable in the plasma as products of cell turn over, particularly from lymphocytes. The serum concentrations of β2M closely depends on renal function because the kidneys are the main site of clearance.

The β2M is a major component in dialysis-related amyloidosis, a disabling disease affecting long-term dialysis patients. Several studies have used β2M as a biomarker in diabetic patients,^8^^-^^11^ but little is known about the advantages of using β2M over other commonly used parameters, such as serum creatinine (Cr) and blood urine nitrogen (BUN) for detection of the kidney disease.

This study was aimed to find interrelationship of β2M, BUN and Cr in streptozotocin (STZ) induced diabetic animals as a model.

## Materials and Methods

**Experimental design. **Twelve New Zealand White rabbits (weighing 1.5-2.0 kg) were randomly assigned into two groups of six and housed in separate cages at 23 ˚C with 12-hour cycles of light and darkness. Food and water were provided *ad libitum*. The animals in treatment group were made diabetic by intraperitoneal (IP) injection of 65 mg kg^-1 ^STZ (Sigma-Aldrich Co., St. Louis, USA) dissolved in citrate buffer with pH 4.5. The control rabbits received the same volume of citrate buffer alone. The development of diabetic state was confirmed by demonstration of fasting blood glucose levels > 200 mg dL^-1^ 24 hr after STZ injection.

**Blood sampling. **Blood samples were collected from the ear marginal veins (3 mL, needle gauge 25) of the all animals of both groups at 24 hr intervals for up to three days, and then every week up to 12 weeks. Sera were isolated from blood samples in tubes with no anticoagulant, after being clotted for 30 min, and centrifuged at 2000 *g* for 5 min. 

**Measurement of serum glucose level. **Serum glucose levels were measured by the glucose oxidase method (GOD- PAP) using a glucose analysis kit (Randox Laboratories Ltd., Crumlin, UK) according to the manufacturer’s instructions.

**Measurement of serum insulin level. **Serum insulin concentration was quantified using a Human Insulin ELISA kit (Millipore, St. Charles, USA), according to the manufacturer’s instructions. 

**Measurement of serum β2M concentrations. **The β2M concentrations of the serum samples were determined using a β2M ELISA Kit (Alpco diagnostics, Salem, USA), according to the manufacturer’s instructions, with few modifications. Briefly, 100 µL of diluted serum (1:10 dilution, Sample Diluent including 2% bovine serum albumin in buffered solution containing preservative) were loaded in duplicate wells in a 96-well plate coated with anti-human β2M monoclonal antibody and incubated at 37 ˚C for 30 min. The wells were washed five times with wash buffer, and 200 µL of horseradish peroxidase-conjugated sheep monoclonal anti-β2M antibody were added into each well. The plate was further incubated at 37 ˚C for 30 min. After five washes with buffer, 200 µL of substrate solution (3,3′,5,5′-Tetramethylbenzidine, TMB) were added into the wells. After a 20 min incubation period in a dark room, the reaction was stopped by adding 100 µL of stop solution (0.5 M sulfuric acid), and the optical density (OD) of yellow color developed was determined within 15 min at 450 nm by using an ELISA plate reader (Denley Instruments Ltd., West Sussex, UK). A reference β2M standard, ranging from 0 to10 µg mL^-1^, was run in parallel with each experiment.

**Measurement of serum BUN and Cr levels. **Serum BUN concentration was determined using a commercial kit (Pars Azmoon Co., Tehran, Iran), according to manufacturer’s instructions. Serum Cr was measured by Jaffe’s method, using a Commercial Kit (Pars Azmoon Co., Tehran, Iran).^12^

**Statistical analysis. **Descriptive statistics were made using SPSS software, (Version 15; SPSS Inc., Chicago, USA). Two-way ANOVA repeated measurements were used to evaluate significant changes between healthy and diabetic groups, and during different days of study respective prior time point in each group. Bonferroni post hoc test was used as a correction for multiple comparison. A *p*-value less than 0.05 was considered as significant. The data are presented as mean ± SD. 

## Results

**Fig. 1:**
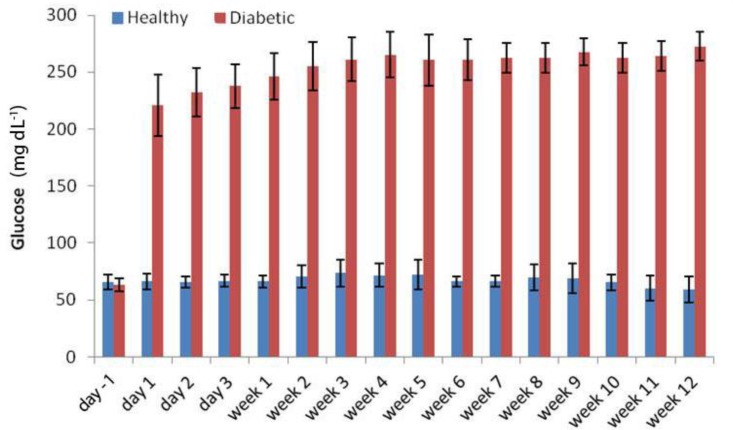
Serum glucose concentrations at different time points. Significant differences between healthy and diabetic groups were observed in all time points after STZ administration. The serum glucose level in diabetic group on day 1 was significantly higher than before injection of STZ

**Fig. 2:**
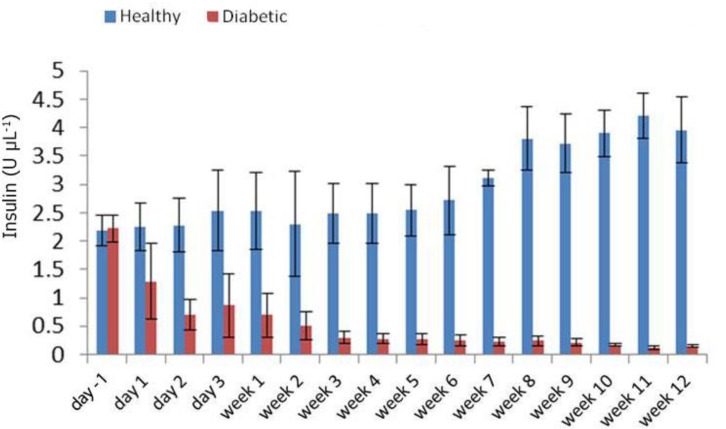
Serum Insulin concentrations at different time points. Apart from before injection and day 1, significant differences were observed between healthy and diabetic groups in other time points

**Fig. 3:**
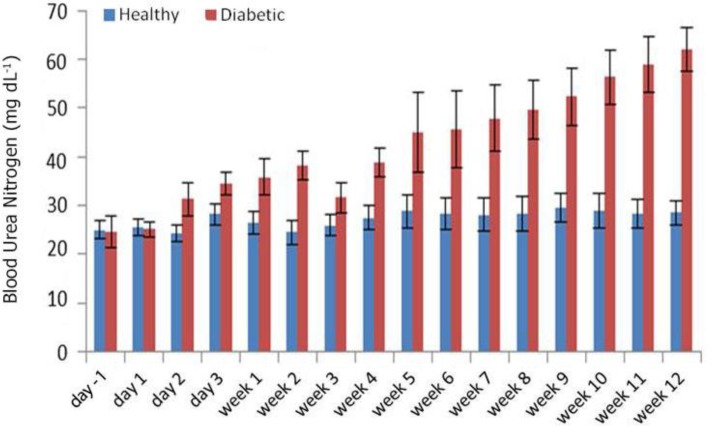
BUN levels at different time points. Significant difference between healthy and diabetic in each time point was started from day 2 onwards. Significant difference in the levels of BUN were observed on day two after STZ administration

Blood glucose concentrations increased 24 hr after STZ administrations and remained high during the course of the Study (Fig. 1). Meanwhile, serum insulin concentrations decreased significantly at day one, and reached their minimum levels 11 weeks after STZ administration (Fig. 2). The BUN concentration in healthy and diabetic groups before injection was 25.00 ± 1.87 mg dL^-1^ and 24.60 ± 3.20 mg dL^-1^, respectively. Except for weeks three and four with a remarkable decrease in BUN, its concentration showed an upward trend of increasing reaching 62.00 ± 4.60 mg dL^-1^ at week 12 in the diabetic group (Fig. 3). 

**Fig. 4:**
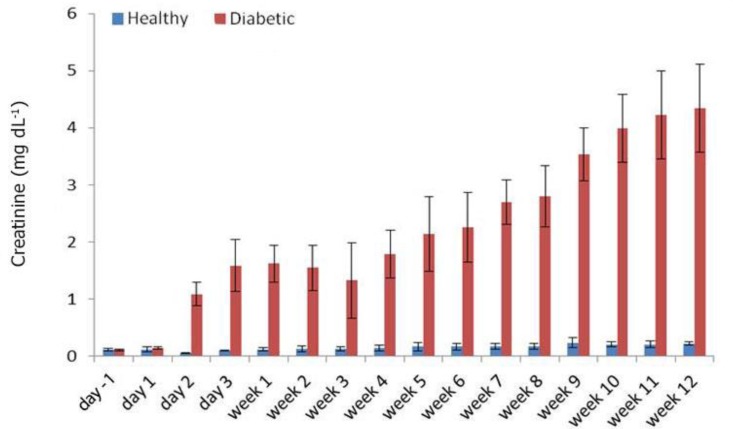
Serum Cr levels at different time points. Significant difference between healthy and diabetic in each time point was started from day 2 onwards. Significant difference in the levels of Cr were observed on day two after STZ administration

**Fig. 5:**
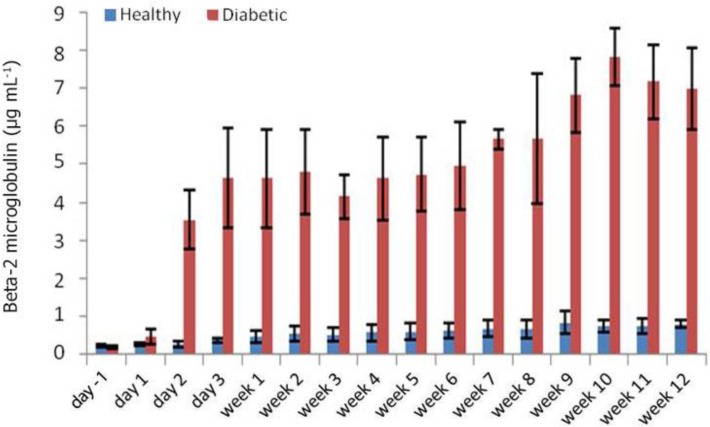
Serum β2M levels at different time points. Significant difference between healthy and diabetic in each time point was started from day 1 onwards. Significant difference in the levels of β2M were observed on day 1 after STZ administration

In diabetic rabbits, a steady increase also observed for the Cr concentrations with increase Cr over time in the serum reaching its highest level of 4.34 ± 0.77 mg dL^-1^ at week 12 and a noticeable decrease at week three (Fig. 4). At day one, the serum level of β2M in diabetic group significantly increased (0.47 ± 0.19 µg mL^-1^) compared with its level before STZ administration (0.18 ± 0.03 µg mL^-1^), (*p* < 0.05). A significant difference was observed between the serum levels of β2M of diabetic group and those of the control ones (0.26 ± 0.04 µg mL^-1^), (*p* < 0.05) at day one post-STZ administration. The serum level of β2M reached 3.54 ± 0.78 µg mL^-1^ at day two in diabetic group and remained high for next nine weeks when at weeks 10 and 11 it showed even a further increase of 7.08 ± 0.75 µg mL^-1^, and 7.16 ± 0.97 µg mL^-1^, respectively whereas in the control rabbits, the level of β2M remained low with no significant changes (Fig. 5).

## Discussion

The normal ranges of glucose and insulin in healthy rabbits were determined in the current study. In the treatment group, serum glucose level gradually increased following administration of SZT to its peak on week 12, this was associated with a gradual decrease in secretion of insulin, which reached its lowest level around week 11 after SZT injection. Significant reduction of serum insulin from day one to a minimal level of (0.12 ± 0.03 U μL^-1^) at week eleven after STZ administrations indicated the development of diabetes mellitus in rabbits of this study.

Streptozotocin is widely used as a diabetogenic agent in animal models to induce diabetic nephropathy. However, it could also be directly cytotoxic to kidneys making it difficult to distinguish between diabetic-related nephropathy and STZ-induced nephropathy.^12^ One study reported that the STZ-induced diabetic rat was not suitable for long-term studies because of progressive renal tumorigenesis.^13^ Additionally, weight loss, respiratory distress, rapid glycemic shifts resulting in life-threatening hypoglycemia, and a generalized poor body condition have already been described as adverse effects of STZ injection.^14^ Nephrotoxicity in the form of transient proteinuria, azotemia, abnormalities of tubular function, and acute renal failure, was described as the major toxic condition following administration of STZ and it has been suggested that squamous metaplasia might be an important part of streptozotocin renal toxicity.^15^ However, Evan *et al*. reported that, in contrast with alloxan, streptozotocin caused no detectable renal injury at the dose 60 mg kg^-1^ which was approximately as the one we used. There was another study stating that no protection procedure was necessary for the kidney when STZ used for inducing diabetes.^16^

In the current study, the serum concentrations of Cr, and BUN were determined as routine tests for kidney function. The BUN concentration except for weeks three and four with a remarkable decrease showed an upward trend of increasing in the diabetic group. The concerned decrease can be explained by an insufficient progress in the regenerative process in the kidney.

In diabetic rabbits, an almost similar pattern to BUN was also observed for the Cr concentrations with a steady increase in Cr over time in the serum reaching its highest level at week 12 and a noticeable decrease at week three. Increased serum levels of BUN, Cr and β2M in diabetic rabbits could be caused by either kidney or none-kidney related factors. In rabbits prerenal azotemia can occur in association with stress, water deprivation, severe dehydration, heat stroke and toxic insults. The rabbit has a limited capacity to concentrate urea and a greater volume of urine is required when urea load increases. It is suggested that urea and Cr levels should be checked on a second sample before making an absolute diagnosis of renal failure in rabbits.^17^ As no histopathological examinations have been performed in the present study, thus it is difficult to associate the elevation of BUN and Cr to a diabetic nephropathy. However, it has been shown that high blood concentrations of urea and Cr in rabbits are usually associated with renal disease and antibody response to some infections.^18^ In clinical diabetic nephropathies, tests for Cr and BUN are routines to confirm the kidney’s involvement and to assess the associated pathology.^19^

Measurements of the levels of β2M in the serum of diabetic rabbits revealed a different pattern to BUN and Cr concentrations. From the second day of STZ injection, the β2M level rose rapidly, up to 6 times, compared to day 1, and increased steadily until week 2. From this time point, it fluctuated slightly until week 3 and then continued to rise to its peak at weeks 10 and 11. 

The β2M has been used as a biomarker for nephropathies.^20^^-^^23^ However, β2M has not been used as a common tool in assessment of diabetic renal involvement. Increased concentrations of β2M have been reported in different studies due to viral infections including human immunodeficiency virus infection, malignancies, and in autoimmune disorders. Increase of β2M in urine at early stage of cadmium exposure has been demonstrated.^24^ Tsuchiya *et al*. showed that β2M in urine is very closely correlated with againg.^25^ Hyperthyroidism is another stimulant of β2M production.^26^ In our study significant increase in β2M, Cr and BUN in diabetic rabbits were observed. The results of our study are indicating the earlier at increase of β2M to those of Cr and BUN suggesting a potential role for β2M as a biomarker in the assessment of possible kidney involvement in diabetes. It seems that irrespective of the root-causes behind rising level of Cr and BUN, β2M is almost always increasing earlier to that of them in diabetic rabbits.

Further studies in animal models are required to determine whether urinary β2M can be considered a valuable diagnostic biomarker in predicting the development of kidney complications in diabetes. 
